# The cypriot blunt-nosed viper *Macrovipera lebetinus lebetinus*: complete mitochondrial genome revealed by next-generation sequencing

**DOI:** 10.1080/23802359.2025.2546948

**Published:** 2025-08-23

**Authors:** Stephan Siegert, Joel Fonseca Nogueira, Daniel Jestrzemski, Mike Reich, Bertram Brenig

**Affiliations:** ^a^ Institute of Veterinary Medicine, University of Goettingen, Göttingen, Germany; ^b^ Universidade Federal do Vale do São-Francisco (Univasf), Petrolina, Brazil; ^c^ Institute of Occupational, Social and Environmental Medicine, Goethe University Frankfurt, Frankfurt, Germany; ^d^ Staatliches Naturhistorisches Museum, Braunschweig, Germany

**Keywords:** Mitogenome, whole genome sequencing, phylogeny, tRNA structure, snake

## Abstract

*Macrovipera lebetinus lebetinus* is the sole venomous snake species endemic to Cyprus, with its population declining due to habitat loss and persecution. The taxonomic status of this species remains uncertain, and there is a significant gap in available genetic information. Here, we present the first complete mitochondrial genome of *M. lebetinus lebetinus*, obtained from two specimens. The mitogenomes (17,151 bp and 17,152 bp) include the standard 37 genes—13 protein-coding genes, 22 tRNAs, and 2 rRNAs—as well as two control regions. Comparative analysis reveals a conserved gene order typical for Viperidae but also an unprecedented structural modification: the complete loss of the D-arm in tRNA-Cys, a feature previously unreported in snakes. Phylogenetic analysis confirms the monophyly of *M. lebetinus lebetinus* and *M. lebetinus schweizeri*. These findings contribute essential genetic data for this under-documented species, providing valuable molecular markers for future phylogenetic and conservation studies.

## Introduction

**Figure 1.**
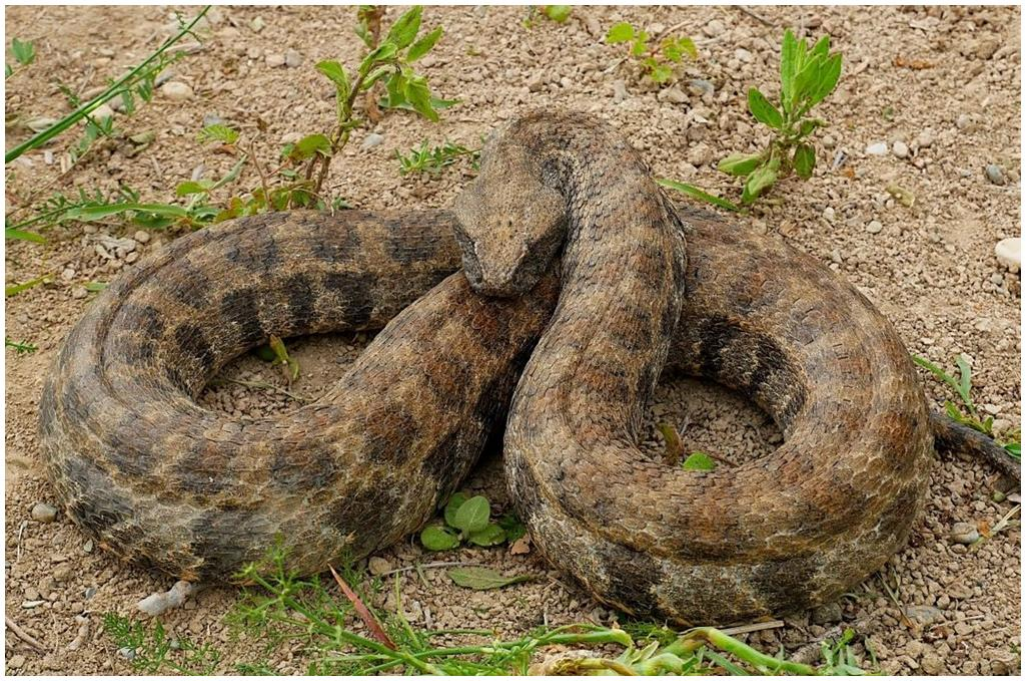
Species reference image of *M. lebetinus lebetinus* (Cypriot blunt-nosed viper). The image shows the characteristic blunt snout and the typical dorsal pattern. Photo: taken by the authors (D. Jestrzemski).

*Macrovipera lebetinus lebetinus*__Linnaeus 1758 (Linnaeus 1758) (Figure 1) is the sole venomous snake species endemic to Cyprus, where it plays an important role in local ecosystems. The species has declined in recent decades, primarily due to habitat loss and widespread human persecution. Surveys indicate that vipers are frequently killed on sight by local residents and hunters, often out of fear or misidentification, which may further threaten the species’ persistence and highlight the need for closer conservation assessment. (Jestrzemski and Kuzyakova 2018; Ilseven et al. 2020)

Although the phylogeny of the genus *Macrovipera (M.*) has undergone several taxonomic revisions (Linnaeus 1758; Herrmann et al. 1992; Lenk et al. 2001; Stümpel 2012; Cattaneo 2020; The Reptile Database 2023), genetic resources for this species remain limited. To date, only the mitochondrial genome of *M. lebetinus schweizeri* has been published (Thanou and Kornilios 2018), leaving a significant gap in the genetic information available for *M. lebetinus lebetinus.*

In this study, we report for the first time the complete mitochondrial genome of *M. lebetinus lebetinus.* The obtained mitogenomes, 17,151 bp and 17,152 bp in length, comprise the typical set of 37 genes – 13 protein-coding genes (PCGs), 22 tRNAs, 2 rRNAs – and features two control regions. Our analysis characterizes the genomic architecture and identifies novel aspects in tRNA secondary structures, which may serve as valuable molecular markers for future phylogenetic and conservation studies.

## Material and methods

### Sample collection and DNA extraction

Two specimens of *M. lebetinus lebetinus* (S 56087 and S 56088) were obtained from the collection of the Staatliches Naturhistorisches Museum (Braunschweig), where they are registered under the accession numbers SNHMB-N.56087 and SNHMB-N.56088. These specimens were originally collected in 2014 on Cyprus (coordinates for S 56087: N 35°01.941′ E 32°27.872′; coordinates for S 56088: N 34°58.938′ E 32°31.795′) and preserved in 80% denatured ethanol.

Approximately 10 mg of tissue per specimen was placed in a heatblock at 56 °C for evaporation of excess liquid. Remaining liquid was removed by centrifugation using a DNeasy Mini Spin Columns (Qiagen, Hilden, Germany). DNA extraction was performed using the DNeasy Blood&Tissue Kit (Qiagen, Hilden, Germany), and quality and quantity of DNA were assessed by agarose gel electrophoresis and NanoDrop spectrophotometer (Thermo Fisher Scientific, Darmstadt, Germany).

### Whole genome sequencing and assembly

Genomic DNA was sheared to approximately 500 bp using a Covaris S220 Focused-ultrasonicator (Covaris, Woburn, MA, USA) and concentrated with Sartorius VIVACON 500 (Fisher Scientific, Schwerte, Germany). Fragment sizes were analyzed on an Agilent 2100 Bioanalyzer (Agilent Technologies, Santa Clara, CA, USA) using the DNA 7500 Kit (Agilent Technologies, Santa Clara, CA, USA). Sequencing libraries were generated using ThruPlex DNA-Seq Kit (Takara Bio, Mountain View, CA, USA) and re-checked on an Agilent 2100 Bioanalyzer before sequencing on a NextSeq 500 (Illumina, San Diego, CA, USA) (300 cycles) using a High Output Flow Cell v2.5 (Illumina, San Diego, CA, USA). For S 56087, 120,724,635 paired-end reads (≈14.7 Gb) and for S 56088, 134,848,237 paired-end (≈18.6 Gb) reads were obtained.

The mitochondrial genomes were assembled by combining reference-guided and de novo approaches using the published mitogenome of *M. lebetinus schweizeri*__Werner 1935 (NCBI accession MH717075) as a reference (Thanou and Kornilios 2018). Assemblies were performed with SeqMan NGen v17.5.0.48 (DNASTAR), Sequencher v5.4.6 (GeneCodes Corporation), and BWA-MEM (Li and Durbin 2009). A coverage depth map is provided in the supplementary materials (Figures S1 and S2).

**Figure 2.**
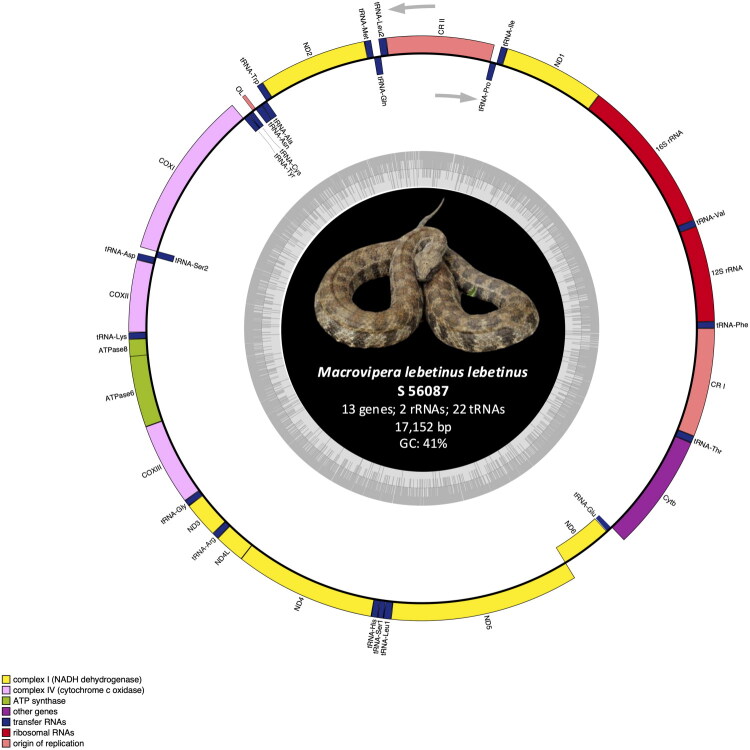
Mitochondrial genome map of *M. lebetinus lebetinus* (S 56087). Genes encoded on the heavy or light strand are respectively indicated on the outside or inside of the circular mitogenome map. Photo: taken by the authors (D. Jestrzemski).

**Figure 3.**
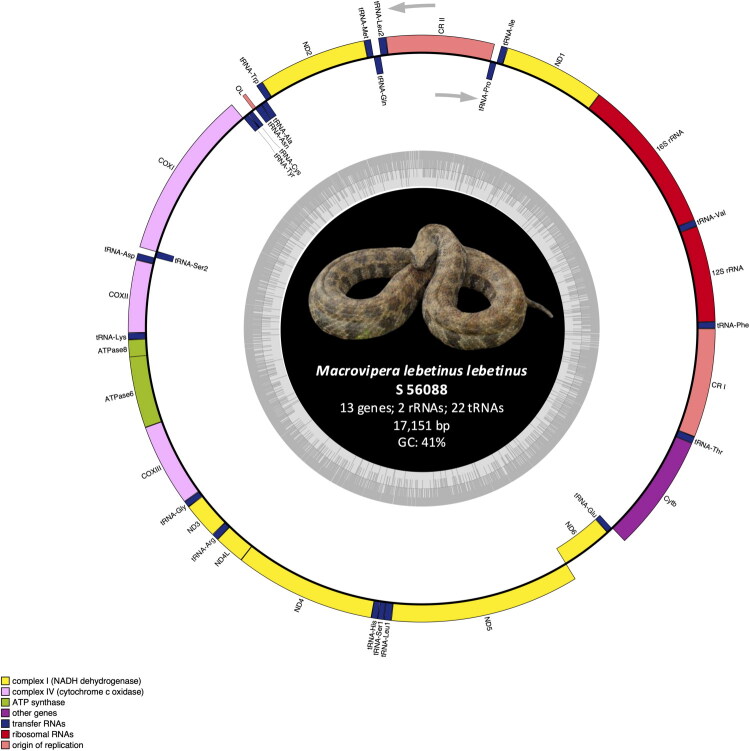
Mitochondrial genome map of *M. lebetinus lebetinus* (S 56088). Genes encoded on the heavy or light strand are respectively indicated on the outside or inside of the circular mitogenome map. Photo: taken by the authors (D. Jestrzemski).

Polymorphic positions within the control regions were resolved by PCR and Sanger sequencing using primers designed with Primer3 (Kõressaar et al. 2018). Primer details are provided in the supplementary materials (Table SI). Figure 3.

### Annotation and phylogenetic analysis

Gene prediction and annotation were performed using MITOS 2.0 (Bernt et al. 2013) and visualized with OGDRAW (Greiner et al. 2019). The secondary structures of 22 tRNA genes were inferred using tRNAscan-SE (Chan and Lowe 2019).

For phylogenetic analysis, complete mitogenomes from eight Viperinae species were obtained from GenBank. Sequences were aligned with MAFFT v7.526 (Katoh and Standley 2013) and analyzed with IQ-TREE v2.4.0 (Minh et al. 2020), employing ModelFinder Plus (Kalyaanamoorthy et al. 2017) for model selection and 1000 ultrafast bootstrap replicates (Hoang et al. 2018). The maximum likelihood tree was visualized using iTOL v6 (Letunic and Bork 2024).

## Results

### Mitogenome sequence structure analysis

The complete mitochondrial genomes of two *M. lebetinus lebetinus* specimens were sequenced and deposited in GenBank under the accession numbers PQ571331 and PQ571332.

The total lengths were determined to be 17,152 bp (S 56087) and 17,151 bp (S 56088), with a nucleotide composition of 32% A, 28% C, 13% G, and 27% T. Both genomes contained the standard 37 genes: 13 PCGs, 22 tRNAs, 2 rRNAs and feature two complete control regions (D-loops) along with a putative L-strand replication origin (OL) (Figures 2 and 3; Supplementary Material – Tables S2 and 3).

The gene pattern conforms to the III-B type arrangement observed in *Viperidae* (Qian et al. 2018) – characterized by duplication of the control region and a rearrangement of tRNA-Leu2. In most vertebrates, tRNA-Leu2 is located between 16S rRNA and ND1; whereas in *M. lebetinus lebetinus* it is shifted between the control region and tRNA-Gln, a configuration common in other *Viperidae* (Hamdan et al. 2023). Moreover, the OL is identical to that reported in *M. lebetinus schweizeri* (Thanou and Kornilios 2018).

### Protein-coding genes and codon usage

The lengths of the PCGs ranged from 165 bp to 1,788 bp. Five different start codons were observed: ATA, ATC, GTG, ATG and ATT. Stop codons included AGA, TAA and AGG, with six genes terminated with incomplete T. These features are consistent with other *Viperidae* mitogenomes, including *M. lebetinus schweizeri* (Thanou and Kornilios 2018; Montaña-Lozano et al. 2023). The higher AT than GC content is typical for reptile mitochondrial genomes (Montaña-Lozano et al. 2023) (Figures 2 and 3; Supplementary Material – Tables S2 and 3).

### tRNAs and rRNAs

All 22 tRNA genes were identified, with lengths ranging from 53 to 73 bp. While most tRNAs adopted to the typical cloverleaf structure, tRNA-Ser1 and tRNA-Cys lacked a dihydrouridine arm (D-arm). The absence of the D-arm in tRNA-Ser1 is a common trait among metazoans (Jühling et al. 2012); however, a complete loss of the D-arm in tRNA-Cys is unprecedented in snakes and has only been reported in certein amphibians, *Tunicata*, *Bryozoa*, *Platyhelminthes*, *Arthropoda*, *Nematoda*, *Lepidosauria* and mammals (Arnason et al. 1997; Macey et al. 1997; Jühling et al. 2012). In *Colubridae*, an abnormal tRNA-Cys lacking the TΨC loop has been documented (Shan and Wang 2022). This appears to be the first report of a complete D-arm loss in tRNA-Cys within *Viperidae*. Despite structural modifications, previous studies suggest that such tRNA remein functional (Jühling et al. 2012). (Supplementary Material – Figure S3) The 12S rRNA and 16S rRNA genes were separated by tRNA-Val, maintaining a conserved arrangement typical for vertebrate mitogenomes (Boore 1999) (Figures 2 and 3; Supplementary Material – Tables S2 and S3).

### Control regions

Two non.coding control regions were identified: CR I was located between tRNA-Thr and tRNA-Phe and CR II was situated between tRNA-Pro and tRNA-Leu2. These structures are conserved within *Viperidae*, although slight variation in length and placement are observed among snake families (Hamdan et al. 2023) (Figures 2 and 3; Supplementary Material – Tables S2 and S3).

### Phylogenetic analysis

The best-fit substitution model selected by ModelFinder was TIM2 + F + I + G4, according to the Bayesian Information Criterion (BIC).

**Figure 4.**
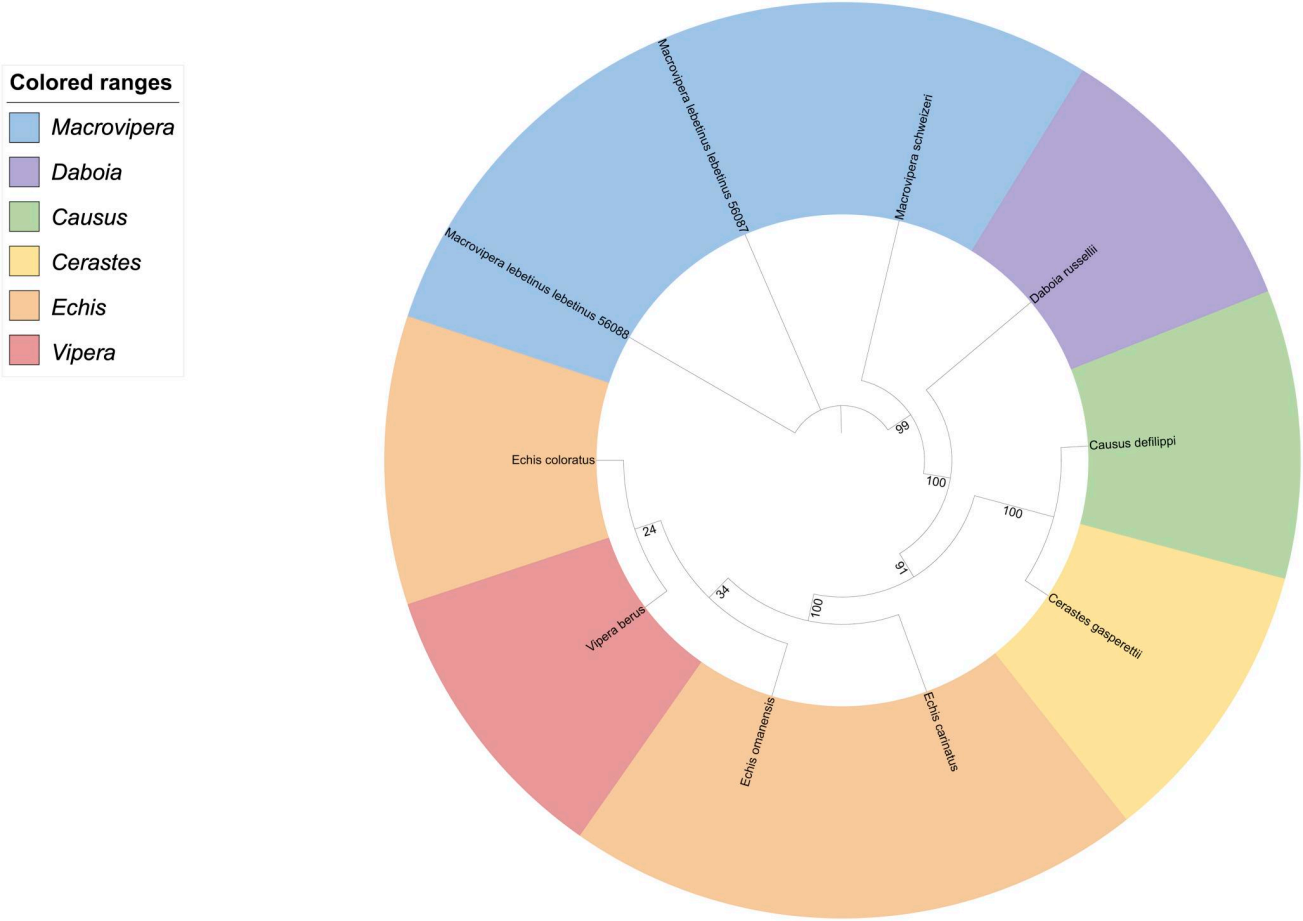
Maximum likelihood tree of complete mitogenomes from Viperinae. Phylogeny considered nine species, including the described mitogenome of *M. lebetinus lebetinus*. GenBank accession numbers and associated references are indicated for each taxon: *Cerastes gasperettii* – CM101945.1 (Mochales 2025); *Echis omanensis* – NC_063589.1 (Khan and Al-Harrasi, 2022); *Echis coloratus* - NC_060592.1 (Khan and Al-Harrasi, 2022); *Echis carinatus* - NC_060591.1 (Khan and Al-Harrasi, 2022); *Macrovipera schweizeri* – NC_044966.1 (Thanou and Kornilios 2018); *Vipera berus* – NC_036956.1 (Gao et al. 2018); *Causus defilippi* – NC_013479.1 (Castoe et al. 2009); *Daboia russellii* – NC_011391.1 (Chen and Fu 2008).

Phylogenetic analysis using nine complete mitochondrial mitogenomes of Viperinae robustly supports the monophyly of *M. lebetinus lebetinus* and *M. lebetinus schweizeri* (bootstrap value 99). The maximum likelihood tree places the genus *Macrovipera* as a sister group to *Daboia* (bootstrap value 100), in agreement with previous studies on Viperidae (Chowdhury et al. 2022) (Figure 4).

Interestingly, the tree also suggests a paraphyletic placement of Echis, with Vipera berus nested within the clade. However, this relationship is poorly supported (bootstrap values <35) and likely reflects limited phylogenetic resolution at this level.

## Discussion and conclusion

This study provides the first complete mitochondrial genome for the persecuted *M. lebetinus lebetinus*. The mitogenomes (17,151 bp and 17,152 bp) encompass the canonical 37 genes and two control regions, exhibiting the typical III-B gene arrangement observed in Viperidae (Qian et al. 2018). Notably, the mitochondrial genome reveals novel features in tRNA secondary structure – specifically, the complete loss of the D-arm in tRNA-Cys – which has not been previously reported in snakes (Arnason et al. 1997; Macey et al. 1997; Jühling et al. 2012).

These findings contribute essential genetic data for *M. lebetinus lebetinus*, enhancing our understanding of its evolutionary history and phylogenetic position within Viperidae. The molecular markers identified here will be valuable for future phylogenetic and conservation studies, particularly in light of ongoing habitat loss and human persecution.

## Supplementary Material

suppl_mat_table_S_III.xlsx

suppl_mat_table_S_II.xlsx

suppl_mat_figure_S_2.png

suppl_mat_table_S_I.xlsx

suppl_mat_figure_S_3.png

suppl_mat_figure_S_1.png

## Data Availability

The genome sequence data that support the findings of this study are openly available in GenBank of NCBI at (https://www.ncbi.nlm.nih.gov/) under the accession no. PQ571331 (for S 56087) and PQ571332 (for S 56088). The associated BioProject, SRA, and BioSample numbers are PRJNA1227756, SRR32478114 (for S 56087), SRR32478113 (for S 56088), and SAMN46992644 (for S 56087), SAMN46992645 (for S 56088) respectively. The raw sequencing chromatograms (AB1 files) used for resolving polymorphisms in control regions I and II are available at Figshare: https://doi.org/10.6084/m9.figshare.28840271.v1.
