# Supplementary figures and images for: The cypriot blunt-nosed viper Macrovipera lebetinus lebetinus: complete mitochondrial genome revealed by next-generation sequencing
Source: Mitochondrial DNA B Resour. 2025 Aug 23;10(9):852–7. doi: 10.1080/23802359.2025.2546948 (PMC12377087; doi:10.1080/23802359.2025.2546948)

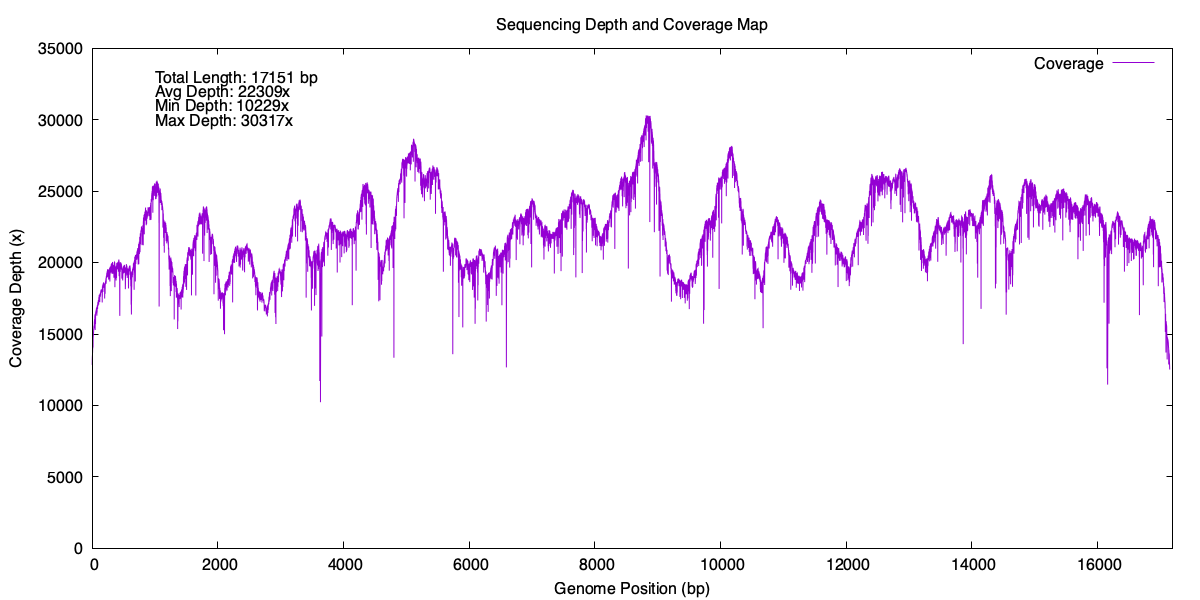

Supplement: suppl_mat_figure_S_2.png [file TMDN_A_2546948_SM8756.png]

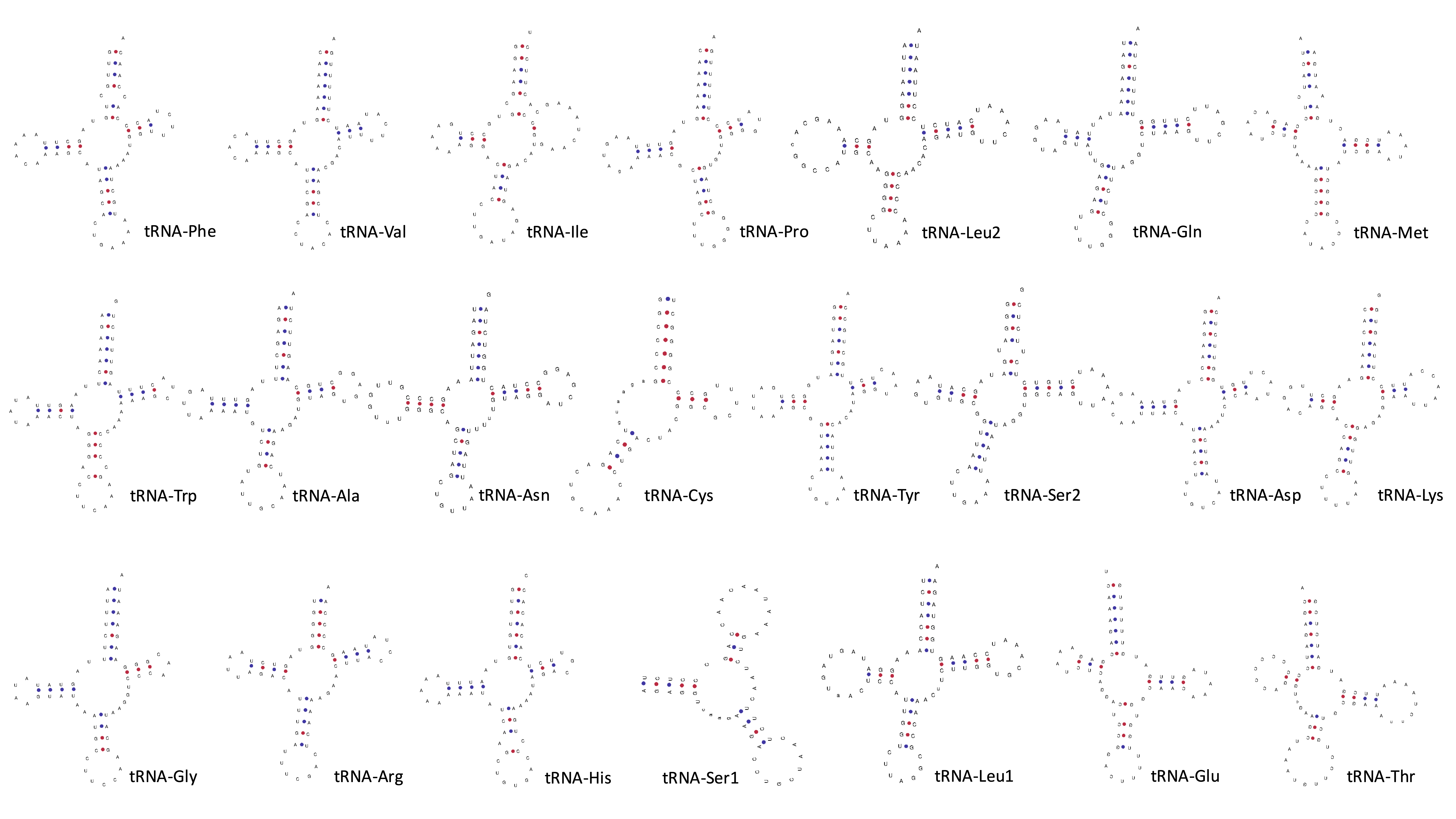

Supplement: suppl_mat_figure_S_3.png [file TMDN_A_2546948_SM8754.png]

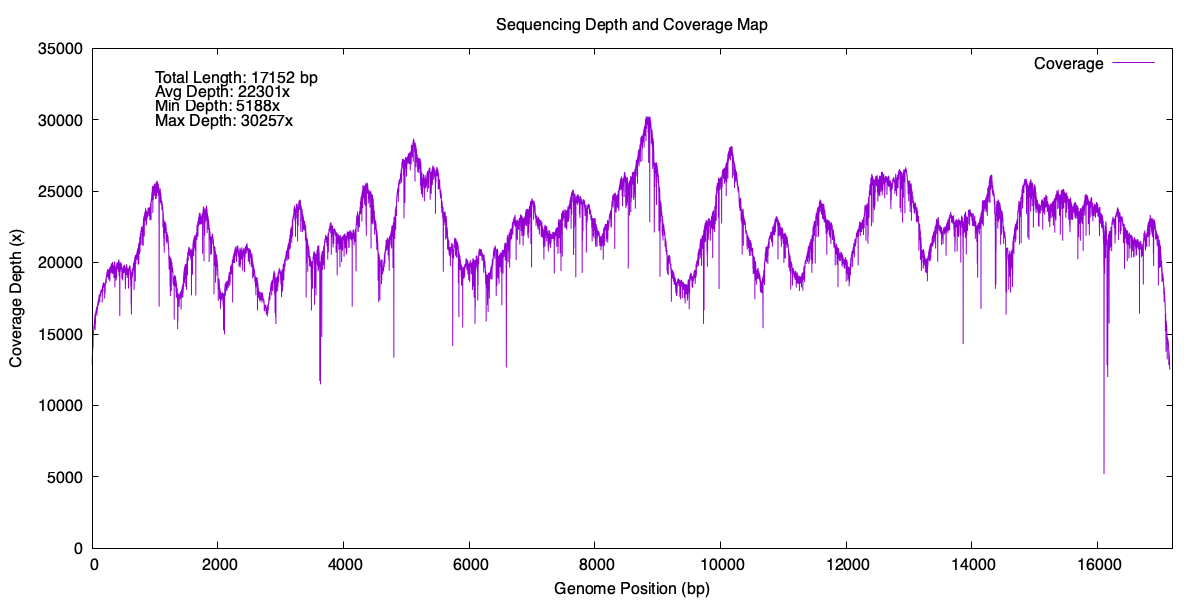

Supplement: suppl_mat_figure_S_1.png [file TMDN_A_2546948_SM8753.png]
